# Health Professionals Facing Burnout: What Do We Know about Nursing Managers?

**DOI:** 10.1155/2014/681814

**Published:** 2014-04-03

**Authors:** Jean-Luc Heeb, Véronique Haberey-Knuessi

**Affiliations:** ^1^Haute Ecole Fribourgeoise de Travail Social, University of Applied Sciences of Western Switzerland, Rue Jean-Prouvé 10, 1762 Givisiez, Switzerland; ^2^Haute Ecole de Santé ARC, University of Applied Sciences of Western Switzerland, Espace de l'Europe 11, 2000 Neuchâtel, Switzerland

## Abstract

*Objective*. To address the degree of burnout in nursing managers in hospitals of Western Switzerland, including comparison with medical managers, and its relationship with personal, work-related, and organizational characteristics. *Methods*. Statistical analysis of the scores of the Maslach Burnout Inventory-Human Services Survey from 257 nursing managers who answered a standardized electronic questionnaire. *Results*. Nursing managers showed a low degree of burnout, which was similar to that of medical managers. Most of them had a low level of emotional exhaustion and a low level of depersonalization, while personal accomplishment was contrasted. Only 2.3% had a high degree of burnout. These findings challenge the hypothesis of high stress being associated with high burnout, as nursing managers can be supposed to have a highly demanding job due to their intermediary position within the hospital hierarchy. Variations of burnout by personal, work-related, and organizational characteristics mainly concerned emotional exhaustion. *Conclusion*. Though nursing managers face a highly demanding job, they may benefit from resources (including coping strategies and empowerment) which help counterbalance job stress. Unequal distribution of resources may play a central role when facing burnout.

## 1. Introduction

Growing evidence has accumulated in the past decades that burnout is a widespread phenomenon among health professionals with adverse consequences on individual health, organizational functioning, quality of care, and patient outcome. Consistent with the hypothesis that high stress results in an increased risk of burnout, research in the field of nursing concentrated on professionals working in demanding settings such as oncology, intensive care, and palliative care. Nursing managers, however, have received very little attention to date, though they accomplish a highly demanding job with both management tasks and work with patients. Furthermore, due to their intermediary position within the hospital hierarchy, they are involved in ongoing hospital reforms, which have been recognized to accentuate the risk of burnout. Thus, scarce evidence and the changing context of the health system in Western countries suggest examining the degree of burnout among nursing managers.

Since Freudenberger's [1] seminal work among health professionals in the 70s, there has been a continuing and growing interest in burnout in the fields of both research and public opinion. Burnout was described as a syndrome referring to mental and physical exhaustion that is rooted in the professional career. Still according to Freudenberger, burnout is likely to appear when professionals are emotionally highly implicated with their patients but the results they expect do not realize, thus curtailing their motivation and causing responses like tiredness, sleepiness, failing to cope, angriness, or cynicism. This view of burnout is mainly individually centred, as it emphasizes the professionals' behaviour and emotions, but it also points to the discrepancy between an idealized self and reality. Therefore, professionals with high expectancies are most at risk to face a “state of fatigue or frustration brought about by a devotion to a cause, a way of life, or a relationship that failed to produce the expected reward” [2, page 13].

While personal investment is central to understanding burnout, conditions of work should also be included [3]. As a result, situational stressors may play a central role when they curtail the sense that the professionals attribute to their work [4]. Recent changes in the health systems may interfere with the meaning and the goals that the health professionals attribute to their work. As in other countries, Swiss public organizations are faced with structural and managerial changes, mainly the introduction of the principles of the new public management, meaning the application of market-oriented management to the public sector which is supposed to increase the efficiency and customer orientation [5]. However, such policies have been shown to be associated with an increased workload and a loss of meaning in the professionals' work. Consequences include intensification of work [6], especially administration [7], diminishing nurse/patient ratio [8], and cross-training of personnel [9]. At the same time, professionals are challenged by demographic changes resulting in higher rates of older patients with multiple pathologies [10, 11].

At the health professionals level burnout is associated with a variety of adverse outcomes ranging from irritability, sleepiness, and depression to self-depreciation or addictive behaviours [12, 13] which may affect the relationship with the patient and result in decreased performance, turnover, and absenteeism at the organizational level [14].

Burnout is most often looked at across the three dimensions of emotional exhaustion, depersonalization, and reduced personal accomplishment [15]. Emotional exhaustion refers to a lack of energy and interest in one's work due to the experience of drained emotional resources. Depersonalization or cynicism manifests in impassive attitudes and thoughts and acts as a protection from exhaustion by making a person distance oneself from others. Finally, a lack of personal accomplishment or self-efficacy reflects feelings of diminished efficacy, competence, and achievement at work. A high level of emotional exhaustion and depersonalization coupled with a reduced sense of personal accomplishment is characteristic of professionals with a high degree of burnout.

To date, studies on burnout among nurses have mainly concentrated on nursing specialities which have been considered to bear a high risk, such as oncology, intensive care, or palliative care [16]. While increased burnout in these specialities may be due to demanding emotional investment with the patients, nursing managers are also faced with a variety of relational tasks including motivation, organization, and conflict resolution with their team to achieve the management policy of the hospital, all in addition to their work with the patient. Indeed, the intermediary position of managers within the hospital hierarchy can be supposed to be a risk factor as hospital restructuring has been shown to increase the degree of burnout [17]. Based on the common view that a highly stressful work environment results in a high degree of burnout [18], nursing managers could be expected to be particularly vulnerable to burnout. This view, however, needs to be questioned in the light of empirical research. Though health professionals are considered to present a high risk of burnout due to their emotionally demanding job, several studies reported low degrees of burnout among nurses [19, 20]. More specifically, there is some evidence that burnout may be lower among nurses belonging to specialities perceived as very stressful than among those working in low-stress settings [21, 22]. Possible explanations for the moderate incidence of burnout may be seen in effective coping strategies [23], including empowerment [24, 25].

A central question therefore is to evaluate the degree of burnout among nursing managers: how do high job demands which are considered as risks factors and possible resources used as protective factors combine? Is burnout among nursing managers similar to nurses without a management function, is it higher, or is it lower? Despite the wide literature about burnout among health professions, studies examining nursing managers are very scarce, with a North American study suggesting a rather low degree of burnout [19]. Thus, the present study aims to contribute to filling this gap by examining the prevalence of burnout and its relationship to personal, work-related, and organizational characteristics in a sample of nursing managers from five Swiss hospitals.

## 2. Methods

### 2.1. Data

Data came from a cross-sectional survey about mental health and job satisfaction among hospital managers in Western Switzerland. The five surveyed hospitals include a university hospital (more than 1000 beds), two regional hospitals (from 300 to 1000), and two local hospitals (less than 300). Data were collected in 2011/2012 anonymously and by means of a standardized electronic questionnaire. It was addressed to all nurses, physicians, and administrative, financial, and technical employees with a manager status. To increase participation the survey was announced by using the internal communication of the hospitals and electronically. In each hospital local referents motivated the managers to participate. A total of 1333 managers were contacted, of whom 942 participated in the survey (nursing managers: 317, medical managers: 226, and other managers: 399). An overall response rate of 70.7% was achieved. Burnout was measured by two different instruments among the health managers and the other managers [15]. Thus, in addition to the subsample of nurses, data from physicians were used to provide comparisons. Due to incomplete data 94 respondents were excluded from the health manager subsample. The final sample consists of 449 nursing managers (257) and medical managers (192).

### 2.2. Instruments

Burnout among nurses and physicians was assessed by the Maslach Burnout Inventory-Human Services Survey (MBI-HSS), a 22-item questionnaire which reflects the dimensions of emotional exhaustion (9 items), depersonalization (5), and lack of personal accomplishment (8). For each item respondents had to indicate the frequency of the job-related feeling described by the item on a 7-point scale ranging from 0 (never) to 6 (every day). To ensure comparison with previous research a high degree of burnout, that is, a high level of emotional exhaustion (score of 27 or higher) and depersonalization (10 or higher) and a low level of personal accomplishment (33 or lower), was defined according to a normative sample of North American nurses and physicians [15].

Personal, work-related, and organizational characteristics were collected in order to address variations in the scores of burnout. Core characteristics include sex, age, length of managerial experience, overtime hours, work schedule, size of the team the manager is responsible for, congruence of personal and organizational values, congruence between personal skills and job requirements, and conflicts within the team. Further characteristics considered were education, household structure, degree of activity, length of overall professional experience, and type of hospital (university, regional, or local).

### 2.3. Statistical Analysis

Analysis focused on nursing managers and was carried out by SPSS 19.0 [26]. Descriptive statistics of the sample characteristics and the MBI-HSS scores are provided. To address variations in the original scores, multivariate linear regression models were computed. Regression analysis concentrates on nursing managers, while descriptive findings include comparison with medical managers.

## 3. Results

### 3.1. Sample Description

The mean age of the surveyed nursing managers was 46.7 years; 58.0% of them were women and they most often lived with a partner and children (56.4%) or alone with a partner (23.3%). They had mainly achieved higher professional or university education (91.5%) and showed longer professional experience (up to 5 years: 8.2%, from 6 to 10 years: 10.9%, from 11 to 15 years: 17.1%, and 16 years or more: 63.8%), including a managerial function (up to 5 years: 30.4%, from 6 to 10 years: 19.5%, from 11 to 15 years: 25.3%, and 16 years or more: 24.9%). The managers were usually responsible for a medium-sized team (up to 10 persons: 13.7%, from 11 to 20: 16.3%, from 21 to 50: 42.8%, and 51 or more: 27.2%). Their degree of activity was rather high (less than 70%: 2.3%, from 70% to less than 90%: 12.5%, and 90% or more: 85.2%). Work schedules were mostly described as regular (79.2%) and overtime hours, though frequent, were not excessive in number (none: 1.6%, from 1 to 10 hours per month: 57.2%, from 11 to 20 hours: 29.6%, and 21 hours or more: 11.7%). Most of the nursing managers worked either sometimes (35.5%) or often (42.2%) with patients. 58.8% were employed in a university hospital, 31.9% in a regional hospital, and 9.3% in a local hospital.

### 3.2. Burnout by Personal, Work-Related, and Organizational Characteristics

Variations of the mean scores of the MBI-HSS subscales by personal and work-related characteristics and variations of the shares of respondents with a high level of emotional exhaustion, a high level of depersonalization, and a low level of personal accomplishment are shown in Table 2. Overall, the variations of the mean scores by the characteristics mirror those of the shares. Most of the variations regard emotional exhaustion with pronounced differences related to work schedules, team size, the congruence of personal and organizational values, and conflicts within the team. Sex and length of managerial experience are associated with depersonalization and overtime with personal accomplishment.

### 3.3. MBI-HSS

Table 1 summarizes the descriptive findings of the MBI-HSS subscales including nursing and medical managers. The mean score of personal accomplishment was significantly lower among nurses than physicians (difference = −1.48, S.D. = 0.66, df = 447, and *P* = 0.023), while no significant difference regarding emotional exhaustion (difference = −0.17, S.D. = 0.71, df = 447, and *P* = 0.808) and depersonalization (difference = 0.02, S.D. = 0.41, df = 447, and *P* = 0.968) was found. Compared with the categorization based on the normative data [15], mean scores of emotional exhaustion were low and those of personal accomplishment average while the mean scores of depersonalization are situated at the threshold between the low and average. Consistent with the mean scores, a small share of respondents had a high level of emotional exhaustion or depersonalization, with a low level being most frequent, and the shares of respondents with a low, average, or high level of personal accomplishment were rather similar.

The simultaneous occurrence of high levels of emotional exhaustion and depersonalization with a low level of personal accomplishment, that is, a high degree of burnout, concerned only a few nursing managers (2.3%; medical managers: 3.1%). The opposite configuration—high personal accomplishment coupled with low emotional exhaustion and depersonalization—was more frequent (12.3%; 14.6%).

### 3.4. Regression Analysis

Table 3 contains the results of the linear regression analysis of the scores of the MBI-HSS subscales. Of the total variance of the subscale scores, the model explained 16.9% for emotional exhaustion, 14.9% for depersonalization, and 5.4% for personal accomplishment. The regression analysis confirms the descriptive findings of Table 2. Increased scores of emotional exhaustion are related to small team size, irregular work schedule, values discrepancy, and, to a minor extent, to young age, perceived lack of fit between personal skills and job demand, or frequent conflicts within the team. Scores of depersonalization were significantly lower among men than women and among managers with longer experience. Finally, personal accomplishment was lower among respondents with numerous overtime hours.

Including further predictors presented in the Methods section such as education, household structure, or the length of overall professional experience into the model did not improve the prediction (result not shown). Only a degree of activity from 90% to 100% (unstandardized coefficient = −1.26, *t* = −1.67, and *P* = 0.096) and working in a local hospital (unstandardized coefficient = 1.70, *t* = 1.91, and  *P* = 0.057) had a borderline effect on the depersonalization score.

## 4. Discussion

Health professionals belong to the traditional occupational groups investigated by the research on burnout. Emotionally demanding tasks, high workload, a stressful environment, or conflicts and unfulfilled expectations when working with patients underpin the view that occupations such as nursing are inherently prone to burnout. Building on this view, research focused on professionals in specialities or settings which are perceived as highly stressful such as oncology, intensive care, or palliative care. Little, however, is known about nursing managers even though they can be presumed to face a very demanding job. Combining both nursing tasks and management tasks like motivation, organization, and conflict resolution may increase the risk of burnout. The present study examined the prevalence of burnout and its variations according to personal, work-related, and organizational characteristics among nursing managers in Western Switzerland.

The main findings of the study concern a low degree of burnout among nursing managers and a widely similar incidence of burnout on nursing and medical managers. Also, characteristics were most often significant predictors for emotional exhaustion, while most of them were not related to depersonalization and personal accomplishment. As regards the degree of burnout, only a small proportion of nursing managers (2.3%) had a high emotional exhaustion, a high depersonalization, and a low personal accomplishment, that is, a high degree of burnout. Though no comparative data are available for nurses without a management function in Switzerland, the proportion of nursing managers is similar to those found for Swiss primary physicians (3.5%; [27]) and medical managers in the present study (3.1%). Compared with North American nursing managers [19], Swiss respondents were consistently less prone to burnout (mean scores of emotional exhaustion: 23.7 versus 16.9, depersonalization: 18.0 versus 5.4, and personal accomplishment: 22.5 versus 35.6). Similarly, a comparison with research on nurses without a manager function in other countries generally shows a similar (nurses in general hospitals [28]) or higher incidence of burnout than for the Swiss nursing managers (oncology nurses [9, 29, 30]). Overall, cross-national comparisons suggest that the rate of nurses with a low level of emotional exhaustion subscale according to the corresponding subscale of the MBI-HSS is particularly low in Switzerland (14.6% according to [31] versus 16.9% in the present study). The lowest rate was found in The Netherlands where, according to another research, human service professionals including nurses showed slightly less favourable mean scores on the three subscales of the MBI-HSS to the present study [32].

There can be several reasons for the low incidence of burnout on nursing managers in Switzerland. Firstly, cultural and contextual differences may explain the higher rates of burnout in the North American manager sample, as nurses tend to have lower scores on burnout in Europe than in North America [32]. Secondly, low degrees of burnout among Europeans might not be specific to managers and reflect a cross-national difference in the prevalence of burnout [31]. Thirdly, in line with theories about coping and empowerment [23–25], managers may benefit from higher resources which are, for instance, related to education or job autonomy to help them face stressful situations than nurses with no management function. Unequal distribution of resources would therefore contribute to explaining differences in the incidence of burnout.

As already pointed out, the proportions of nursing managers and medical managers with a high degree of burnout are similar in the present study. Mean scores on the emotional exhaustion and the depersonalization subscales did not differ significantly, while personal accomplishment was only slightly lower among nursing managers. To our knowledge, research has not yet compared nursing and medical managers. Findings from studies about nurses and physicians do not consistently indicate which ones are more prone to burnout [29, 30]. A possible explanation for the similarity of burnout between nurses and physicians in the present study may be due to the converging tasks and hierarchically comparable positions. For instance, subordination of nurses to physicians may be less accentuated in the case of managers and specific attributes of nurse and physician activities may play a minor role compared to the similar management tasks of both groups.

Prediction of burnout was contrasted as the personal and work-related characteristics were mainly related to emotional exhaustion. While job-related characteristics are classical predictors of burnout, they were only partly associated with burnout in the present study [30]. As an indicator of workload, overtime reduced the scores of personal accomplishment, but it was not related to the two other subscales. Irregular working hours and frequent conflicts within the team strongly accentuated the emotional exhaustion. Personal characteristics like sex, age, and managerial experience showed a moderate association with burnout. Scores were lower for younger age (emotional exhaustion) and longer experience (depersonalization), while depersonalization was more pronounced among women than men. Results for age and experience are widely consistent with previous research [33], while findings regarding sex are variable [34]. Increased emotional exhaustion was also related to discrepancies between personal and organizational values and between personal skills and job demands, thereby emphasizing the role of perceived work conditions. These results may be related to the homogeneity of the respondents due to their management function in the present study. It may be speculated that they encounter widely similar work constraints. Homogeneity could then result in less variations across the characteristics under study, especially for the dimensions of burnout related to the relationships with other persons (depersonalization) and to efficacy at work (personal accomplishment). Emotional exhaustion, however, varies across the characteristics, revealing a possible association with personality features [35].

The present study has some limitations. Firstly, as participants were contacted at their work place, those who were absent for a long period could possibly not be reached. Therefore, the study probably underestimates the proportion of health managers with a high degree of burnout, as they are likely to be on sick leave. Though this recruitment bias is common to most studies about burnout in organizational settings, findings should be considered to be conclusive for rather moderate ranges of burnout. Furthermore, the comparison of burnout between studies may be uncertain as the share of nonsurveyed professionals with high scores may vary across the settings investigated by the different studies. Secondly, findings should not be interpreted in a clinical sense as the self-reports obtained by the MBI-HSS are not diagnostics. In particular, the categorization of burnout based on the normative North American sample is merely a statistical one, as it classified the respondents on a distributional criterion (lower, middle, and upper third of the scores [15]). Categorization is thus arbitrary and depends on a specific sample which is ignoring, for instance, cultural differences between countries, and even slight shifts in the thresholds may lead to strong changes in burnout rates [32]. It should not be viewed as being intrinsically tied to burnout but rather as a common basis to draw comparisons across settings. Hence, the present study widely analysed the scores of burnout themselves in addition to its categorization. Thirdly, as participation in the study was on a voluntary basis and characteristics of nonparticipants could not be identified, some subgroups may be over- or underrepresented. Also, contextual characteristics such as cultural factors or specificities of the health system were not included in the analysis. Thus, generalizations should be made carefully, especially when using the results of the present study for cross-national comparisons.

## 5. Conclusion

Though nursing managers could be thought to be especially at risk due to their demanding job, they showed a rather low degree of burnout in the present study. Therefore, the issue of whether high stress settings lead to high burnout should be questioned further. Differences in resources including coping strategies between nurses with and without management function should be investigated.

## Figures and Tables

**Table 1:** Mean scores and categorization of burnout according to the MBI-HSS subscales among nursing managers (medical managers).

Subscale	Mean score [S.D.]	Low level	Average level	High level
Emotional exhaustion	16.9 [7.1] (17.1 [8.0])	61.1% (54.7%)	28.0% (32.8%)	10.9% (12.5%)
Depersonalization	5.4 [4.2] (5.4 [4.3])	53.7% (57.3%)	29.6% (23.4%)	16.7% (19.3%)
Personal accomplishment	35.6 [6.9] (37.1 [6.6])	35.8%(29.7%)	35.8% (32.3%)	28.4%(38.0%)

Data of medical managers are indicated in parenthesis.

**Table 2:** Mean scores and prevalence of burnout according to the MBI-HSS subscales among nursing managers.

	Mean scores [S.D.]			Shares		
	EE	DP	PA	High EE	High DP	Low PA
Sex						
Men	17.2 [6.8]	6.9 (4.2)	35.7 (6.9)	10.2%	25.9%	36.1%
Women	16.7 [7.3]	4.3 (3.9)	35.6 (6.9)	11.4%	10.1%	35.6%
Age						
Up to 40 years	17.5 [7.1]	5.4 (4.3)	35.2 (7.1)	13.0%	17.1%	38.9%
41 years or more	15.3 [6.8]	5.4 (4.0)	36.9 (6.1)	4.7%	15.6%	26.6%
Managerial experience						
Up to 10 years	17.0 [7.1]	6.0 (4.2)	36.1 (6.4)	11.7%	21.9%	33.6%
11 years or more	16.8 [7.1]	4.9 (4.2)	35.1 (7.3)	10.1%	11.6%	38.0%
Overtime						
Up to 20 hours/month	16.8 [7.1]	5.5 (4.3)	35.2 (7.0)	6.7%	16.7%	20.0%
21 hours/month or more	17.6 [7.1]	5.2 (4.2)	38.6 (7.3)	11.5%	16.7%	37.9%
Work schedule						
Irregular	17.2 [7.1]	5.4 (4.2)	35.7 (6.9)	11.7%	15.9%	35.1%
Regular	14.0 [6.8]	5.6 (4.5)	34.6 (7.2)	0.0%	27.8%	44.4%
Team size						
Up to 5 persons	21.3 [6.5]	6.3 (3.9)	33.9 (5.6)	18.2%	18.2%	45.5%
6 persons or more	16.5 [7.0]	5.3 (4.2)	35.8 (7.0)	10.2%	16.6%	34.9%
Personal/organizational values fit						
Yes	15.7 [6.8]	5.3 (4.3)	36.4 (5.8)	9.0%	16.9%	35.0%
Partly or no	20.0 [7.4]	6.0 (4.1)	35.6 (7.1)	17.2%	19.0%	34.5%
Skills/demand fit						
High	16.2 [7.1]	5.3 (4.3)	36.2 (7.1)	9.0%	15.7%	32.5%
Medium or low	18.9 [7.2]	5.9 (4.2)	34.7 (7.0)	17.5%	22.8%	42.1%
Team conflicts						
Frequent	18.3 [7.5]	6.2 (4.5)	35.8 (7.0)	20.6%	20.6%	30.2%
Seldom or none	16.3 [7.1]	5.1 (4.2)	35.8 (6.8)	7.5%	16.3%	36.9%

EE: emotional exhaustion, DP: depersonalization, and PA: personal accomplishment.

**Table 3:** Linear regression model of the MBI-HSS subscales among nursing managers.

	EE			DP			PA		
	Unstandardized beta coefficient	*t*	*P* value	Unstandardized beta coefficient	*t*	*P* value	Unstandardized beta coefficient	*t*	*P* value
Constant	22.24	15.42	0.000	8.77	10.12	0.000	34.89	23.30	0.000
Men (women)	0.74	0.87	0.384	2.81	5.54	0.000	0.11	0.13	0.896
Age ≤40 years (>40 years)	2.66	2.36	0.019	1.01	1.49	0.138	1.49	1.27	0.205
Managerial experience ≤10 years (>10 years)	1.35	1.36	0.174	1.73	2.90	0.004	0.36	0.35	0.727
Overtime ≤20 hours per month (>20 hours)	−1.39	−1.04	0.300	−0.38	−0.47	0.640	3.18	2.29	0.023
Irregular work schedule (regular)	4.54	2.72	0.007	0.03	0.03	0.973	−2.22	−1.28	0.201
Team size ≤5 persons (>5 persons)	5.14	3.39	0.001	1.06	1.16	0.246	−1.16	−0.73	0.463
Personal/organizational values fit: yes (partly or no)	−3.79	−3.64	0.000	−0.78	−1.24	0.216	−0.91	−0.84	0.402
High skills/demand fit (medium or low)	−2.05	−1.98	0.049	−0.41	−0.66	0.510	1.62	1.50	0.135
Frequent team conflicts (seldom or none)	1.99	2.01	0.046	0.88	1.48	0.139	−0.13	−0.13	0.900

EE: emotional exhaustion, DP: depersonalization, and PA: personal accomplishment.

Reference groups are indicated in parentheses.
